# Action of 2,6-Dichloro-1,4-benzoquinone on the O_2_-Evolving Activity of Photosystem II in *Chlamydomonas reinhardtii* Cells with and without Cell Wall: Inhibitory Effect of Its Oxidized Form

**DOI:** 10.3390/cells12060907

**Published:** 2023-03-15

**Authors:** Vasily V. Terentyev, Anna K. Shukshina, Angelina A. Chetverkina

**Affiliations:** Institute of Basic Biological Problems, FRC PSCBR RAS, 142290 Pushchino, Russia

**Keywords:** *Chlamydomonas*, cell wall, 2,6-dichloro-1,4-benzoquinone (DCBQ), photosystem II, O_2_ evolution rate

## Abstract

*Chlamydomonas reinhardtii* is a widely used object in studies on green algae concerning both photosynthesis aspects and possible biotechnological approaches. The measurement of the maximum O_2_ evolution by photosystem II (PSII) in living algal cells in the presence of artificial acceptors is one of the commonly used methods for determining the photosynthetic apparatus state or its change as compared to a control, parent strain, etc., because PSII is the most sensitive component of the thylakoid membrane. The present study shows the need to use low concentrations of 2,6-dichloro-1,4-benzoquinone (DCBQ) paired with potassium ferricyanide (FeCy) for achieving the maximum O_2_ evolution rate, while a DCBQ concentration above certain threshold results in strong suppression of O_2_ evolution. The required DCBQ concentration depends on the presence of the cell wall and should be exactly ~0.1 mM or in the range of 0.2–0.4 mM for cells with and without a cell wall, respectively. The inhibition effect is caused, probably, by a higher content of DCBQ in the oxidized form inside cells; this depends on the presence of the cell wall, which influences the efficiency of DCBQ diffusion into and out of the cell, where it is maintained by FeCy in the oxidized state. The possible mechanism of DCBQ inhibition action is discussed.

## 1. Introduction

The photosynthetic processes occurring on the thylakoid membrane are coupled with the rapid photoinduced transport of electrons taken from water molecules across the electron transport chain (ETC). ETC includes large transmembrane pigment-protein complexes of photosystem II (PSII), containing the water-oxidizing complex (WOC), cytochrome *b_6_f*, and photosystem I; and small electron carriers such as membrane-associated plastoquinol (PQH_2_; a reduced and protonated form of plastoquinone (PQ)), lumenal water-soluble, copper-containing protein plastocyanin (or cytochrome *c*_6_), as well as stromal iron-sulfur protein ferredoxin and ferredoxin:NADP^+^ oxidoreductase [1]. 

To evaluate the maximal photosynthetic activity of PSII separately from other ETC participants in the case of algal cells, isolated chloroplasts or thylakoid membranes (or in the case of PSII particles), various artificial acceptors capable of taking electrons effectively from the PSII acceptor side have to be used. Applying acceptors should remove the limitation of electron transport within the reaction center of PSII, which can suppress the water oxidation reaction in the WOC and, thus, the O_2_-evolving activity of PSII. The chemical nature of the acceptors used depends on the goal of the study, while benzoquinone derivatives alone or paired with potassium ferricyanide (FeCy) are commonly used [2,3,4,5,6]. As considered; a molecule of benzoquinone derivative is able to replace a molecule of the native mobile secondary quinone acceptor; PQ B (Q_B_); in the Q_B_ site (see; e.g., [4,7]) and thus accepts electrons directly from the PSII primary quinone acceptor; PQ A (Q_A_). FeCy is needed to keep the molecules of benzoquinone derivatives in oxidized form (see; e.g., [2,8]). 

Lipophilic 2,6-dichloro-1,4-benzoquinone (DCBQ) is one of the most commonly used artificial acceptors in studies of the O_2_-evolving activity of PSII in subchloroplast preparations isolated from higher plants, algae, and cyanobacteria [3,5,9,10,11]. DCBQ is also often used for the measurement of the O_2_-evolving activity of PSII in living cells of green algae, including *Chlamydomonas reinhardtii* [12,13,14,15,16]. The small size and lipophilic nature of a DCBQ molecule allow it to easily penetrate the lipid membranes of algal cells, including plasma membranes and membranes of chloroplasts and reach and interact with the acceptor side of PSII in thylakoid membranes. 

In the last decade, the property of DCBQ molecules to be electron shuttles between the PSII acceptor side (or PQ pool) and a collecting (external) electrode has been widely used in research related to electricity production with PSII [17,18,19,20]. In such studies, an electrochemical set-up is more often filled with living green algal suspension, for example, *C. reinhardtii* [17,18,19]. It was proposed that the diffusion efficiency of DCBQ molecules through different lipid membranes of algal cells can be one of the rate-limiting factors in the transfer of electrons from PSII [17]. Recently, the authors [18] tried to calculate the time for quinones to cross a lipid membrane of ~4 nm, which is close to the thickness of the thylakoid membrane [21] and the aqueous compartments of an algal cell with a 3 µm radius. The obtained value was ~180 ms [18]. However, in the case of *C. reinhardtii* cells, a cap-shaped chloroplast located about three times closer (within ~1 µm) to the plasma membrane [22], and PSII complexes are mainly found in thylakoid stacks (grana), which may include about 3–5 thylakoids [23], i.e., 6–10 parallel lipid membranes.

The electron transfer from Q_A_ to Q_B_, which is the slowest step in PSII, takes 200–800 µs [1]. This means that a DCBQ molecule can be reduced by PSII twice in a 2 ms range, which is 50–100 times faster than the proposed single-DCBQ-molecule diffusion rate from an algal cell to outside of it, where DCBQ can be oxidized by FeCy or an electrode. Indeed, the enhancement of photosynthetic electron flow from a *C. reinhardtii* cell suspension to an external electrode was observed due to an increase in DCBQ concentration in the micromolar range (up to 200 µM) [18]. In addition, the stimulation of the O_2_ evolution rate obtained by increasing the DCBQ concentration (up to 300–400 µM) has been previously shown for PSII preparations isolated from higher plants, green algae, and cyanobacteria [3,6,24,25,26].

At the same time, the increase in DCBQ content above the saturation point (even in a 1 mM range) leads to clear inhibition of the O_2_-evolving activity of PSII, which has been shown for subchloroplast preparations from different photosynthetic organisms [3,6,24,25,26]. The exact explanation of this effect is still under debate. In experiments with modified PSII from spinach that lost all proteins and the inorganic core of the WOC, the involvement of DCBQ in electron transfer from reduced species to the primary (P_680_) or secondary (Tyr_z_) electron donors was proposed [8]. However, the possibility of such cycle electron transport in native PSII, which would suppress WOC function, remains questionable. Other results obtained with PSII preparations from cyanobacteria propose the PQ pool (PQH_2_ molecules) as a second site for DCBQ to accept electrons [6], but the *K*_m_ value of DCBQ for the Q_B_ site should be relatively small (i.e., it should have high affinity) compared with those for the PQ pool [27].

The inhibitory effect of increased DCBQ content on the O_2_ evolution rate in living cells of green alga *C. reinhardtii* has not been investigated yet. At the same time, the DCBQ concentrations used during O_2_ evolution measurements vary in a wide range of values, from 250 µM to 1 мМ [12,13,14,15,16]. The possible suppression of the O_2_-evolving activity of PSII in *C. reinhardtii* cells due to increased DCBQ concentration can lead to obtaining incorrect results of the maximum O_2_ evolution rate in studies with algal cultures. 

Additionally, the strains of *C. reinhardtii* used can be either with or without a cell wall, which has a complicated structure. Its five layers (W1, W2, W4, W6 and W7, from inside to outside the cell) have different physical and chemical properties, with varied combinations of proteins, glycoproteins (many of which are highly enriched in hydroxyproline), and carbohydrates, as well as with different stacking schemes of components [28,29]. For example, layers W4 and W6 have the property of dissolving in the presence of chaotropic salts, while layer W2, on the other hand, is a non-crystalline, highly insoluble framework [29]. The thickness of the cell wall in *C. reinhardtii* is less than 0.2 µm; however, it is obvious that it can strongly change the efficiency of the DCBQ molecule exchange between the surrounding medium and the intracellular space. 

In the present study, the influence of the DCBQ concentration in the presence, as well in the absence, of FeCy on the O_2_-evolving activity of PSII in living cells of *C. reinhardtii* was studied. The inhibitory effect of increased DCBQ concentrations was observed; this depended on the presence of the cell wall, which probably decreased the DCBQ exchange between the intracellular space and the surrounding medium, thus changing the ratio between its oxidized (DCBQ_ox_) and reduced (DCBQ_red_) forms to the latter. The obtained data allow us to suppose the involvement of the oxidized form of DCBQ in the inhibitory effect.

## 2. Materials and Methods

### 2.1. Strains and Growth Conditions

The two strains of *C. reinhardtii* studied in the present work were the cell wall-less mutant CC-503 cw92 mt+, taken as the standard wild type (WT) in the series of previous work [3,22,30,31,32], and the basic 137c (CC-125 mt+) WT. Cultures were grown photoautotrophically in parallel in the minimal medium at 25 °C under aeration with air enriched with 5% CO_2_. Continuous illumination was provided with cool white LED lamps at 90–100 µmol photons m^–2^ s^–1^. 

### 2.2. Confocal Microscopy

Confocal microscopy was performed with Leica TCS SPE (Leica Microsystems CMS GmbH, Mannheim, Germany) using a 60 × oil-immersion objective. Cells were immobilized according to a previously described method [33] by rapidly mixing 5 μL of cell suspension with an equal volume of molten 1% low-melting-temperature agarose cooled to 40–45 °C. A volume of 2–5 µL of this mixture was immediately transferred on a cover slip, which was inverted on a microscope slide and allowed to solidify. For cell wall staining, cell suspensions, before immobilization, were mixed with an equal volume of Calcofluor^®^ White M2R (Thermo Fisher Scientific, Waltham, MA). Chlorophyll (Chl) autofluorescence was detected with the following parameters: excitation by laser at 635 nm and registration in the 640–750 nm range. The fluorescence of Сalcofluor white was detected with the following parameters: excitation by laser at 405 nm and registration in the 417–520 nm range. Simultaneous transmission images of cells were obtained.

### 2.3. Oxygen Evolution Measurements

The rate of photosynthetic oxygen evolution by PSII in living algal cells was measured at 25 °C with a Clarke-type electrode in a 1 mL cell (Hansatech Instruments Ltd., Norfolk, UK), using DCBQ and FeCy as artificial electron acceptors and illumination with red light (≥600 nm) at saturation intensity of 2040 µmol photons m^–2^ s^–1^. Before measurements, cell suspensions were diluted with growth medium to a Chl concentration of 10 µg mL^–1^.

### 2.4. Chlorophyll Concentration

The Chl concentration was determined spectroscopically after the extraction of pigments in 80% acetone [34].

### 2.5. DCBQ Reduction Measurements

In order to observe the light-induced reduction in oxidized DCBQ in the presence of algal cells, 30 µM of DCBQ dissolved in dimethyl sulfoxide was added to 1 mL of cell suspension with a Chl concentration of 10 µg mL^–1^ in a growth medium. Absorption changes at 230–350 nm were recorded in a 1 mL quartz cuvette before and after DCBQ addition, leading to the appearance of a peak at 272–273 nm, as well as at different time points after sample illumination with red light (≥600 nm) at 2040 µmol photons m^–2^ s^–1^, leading to the disappearance of the peak, which indicated DCBQ reduction [35]. 

For ΔA_272_ calculation, the difference at 272 nm between the absorption peak and the point on the straight line drawn between the peak ends at ~240, and ~300 nm was used. The value obtained after DCBQ addition was taken as 100%.

### 2.6. Statistical Analysis

Statistical analysis was performed using the standard algorithms of OriginPro (version b9.3.226, 2016) (OriginLab, Northampton, MA, USA). The data are presented as means ± SDs.

## 3. Results

### 3.1. Presence of Cell Wall 

**Figure 1:**
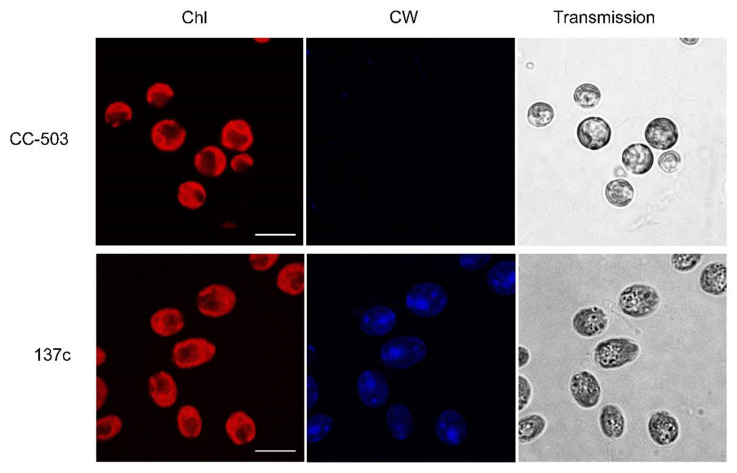
Transmitted images of algal cells obtained with confocal microscopy for determination of Chl autofluorescence (Chl) and fluorescence of Calcofluor white (CW). CC-503 and 137c are strains of *С. reinhardtii* without and with a well-formed cell wall, respectively. The scale bar is 10 µm.

Two commonly used strains of *С. reinhardtii*, CC-503, and 137c (CC-125) were taken as a well-known wall-deficient mutant and its background WT with a well-formed cell wall, respectively. To be sure of the reduction in the cell wall in the case of CC-503, the cell suspensions were stained with a fluorescent blue dye, Calcofluor white, which interacted with the polysaccharides of the cell wall, and analyzed with confocal microscopy. As shown in Figure 1, the shape of the algal cells was different between the two strains. In the transmitted images, CC-503 cells were clearly rounded, while 137c cells were oval. According to known data [36], the rounded shape of *C. reinhardtii* cells could be one of the reasons for the absence of the cell wall. The fluorescent signal of Calcofluor white dye was not detectable in the case of CC-503 cells, while it was clearly observed in 137c cells, which is consistent with previously published results [37]. Thus, the cells of the CC-503 strain used in this study did not, in fact, have a cell wall, in contrast to cells of the 137c strain.

At the same time, according to the Chl autofluorescence results, the cells of both strains had usual, well-formed, cap-shaped chloroplasts with well-defined lobes and basal regions with darkened cycles related to the pyrenoid. Indirectly, this can testify to non-stressed growth conditions for algal cultures. For example, it was previously observed that high light intensity should lead to the reduction in lobes and the disappearance of pyrenoids in *C. reinhardtii* cells [22,23,38].

### 3.2. Influence of DCBQ on the O_2_ Evolution Rate 

**Figure 2:**
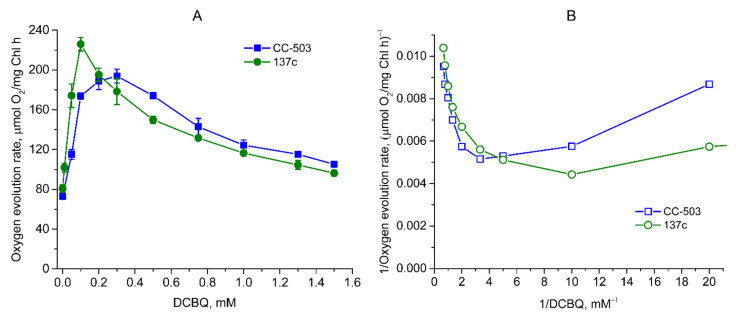
Dependence of O_2_ evolution rate on DCBQ concentration in the presence of 0.5 mM FeCy observed in living cells of CC-503 (blue squares) and 137c (green circles) strains of *C. reinhardtii*. The results are presented as normal (**A**) and double-reciprocal (**B**) plots. The average values of A were used to calculate the values of B. SDs (*n* = 5) are shown as bars in A.

The measurement of the O_2_ evolution rate in living cells of *C. reinhardtii* in the presence of 0.5 mM FeCy (as was previously used [2,15]) showed an increase in its value by 156–166% when using the range of DCBQ concentrations from 0 to 0.2–0.3 mM in the case of CC-503 (Figure 2A). An increase in the O_2_ evolution rate in a similar manner was also observed in 137c cells, but the maximum value was already reached at 0.1 mM DCBQ, with an increase by ~179% as compared to the value obtained at 0 mM DCBQ. The shapes of the peaks on the obtained graphs related to the cells of the CC-503 and 137c strains differ significantly (Figure 2A). It can be seen that the peak is wide for CC-503 cells, with close results within the 0.2–0.3 (probably up to 0.4) mM DCBQ range. At the same time, for 137c cells, the peak is narrow, with a clear maximum at 0.1 mM DCBQ. At 0.2 and 0.3 mM DCBQ, the O_2_ evolution rate already decreased by 14% and 21%, respectively, compared with that obtained at 0.1 mM DCBQ, in contrast to that observed in CC-503 cells.

Following a further increase in DCBQ concentrations above a certain threshold, leading to the induction of the maximum evolving activity of PSII in *C. reinhardtii* cells, strong suppression of the O_2_ evolution rate was clearly detected in both strains as can be seen in Figure 2A, in the plot with absolute values, the two curves of O_2_ evolution decrease in a quite similar manner. At the same time, the curve for 137c cells has a stronger decline than that for CC-503 cells. Indeed, 50% of the maximum value induced by DCBQ addition was reached in 137c cells at ~0.5 mM DCBQ, while in CC-503 cells, this was only reached at ~0.9 mM DCBQ (Figure 2A). Interestingly, when taking into account the absolute values of the plot, then both CC-503 and 137c cells lost a similar portion of the total O_2_-evolving activity equal to ~31–32% at the above-mentioned DCBQ concentrations. At 1 mM DCBQ, the losses of induced O_2_ evolution in CC-503 and 137c cells were ~58% and ~76% of the maximum value, respectively, while at 1.5 mM DCBQ, these were already ~73% and ~90%, respectively.

Thus, the presence of the cell wall somehow leads to the achievement of maximum O_2_ evolution with 2–3 times lower DCBQ concentrations, with more pronounced suppression of O_2_ evolution with the further increase in DCBQ concentrations.

The recalculation of the obtained data on the dependence of the O_2_ evolution rate on DCBQ concentration using double-reciprocal plots resulted in non-straight lines for the cells of both strains (Figure 2B). This indicates that DCBQ could accept electrons not only directly from Q_A_ in the Q_B_ site but also at other locations, which, as previously suggested, could be the molecules PQH_2_ (PQ pool) [6]. 

**Figure 3:**
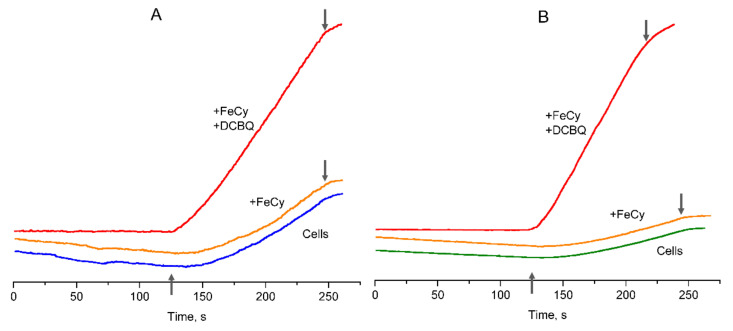
Curves of O_2_ evolution observed in the suspension of living cells of *C. reinhardtii* strains: CC-503 (**A**) and 137c (**B**). Blue and green (CC-503 and 137c, respectively) lines correspond to cells without additions, orange lines refer to cells in the presence of 0.5 mM FeCy, red lines refer to cells in the presence of 0.5 mM FeCy and 0.2 mM or 0.1 mM DCBQ in the case of CC-503 or 137c cells, respectively. ↑ and ↓ indicate approximate moments when the actinic light was switched on and switched off.

The algal cells without additions showed a slightly delayed induction of O_2_ evolution after actinic light was switched on (Figure 3), with the achievement of the maximum O_2_ evolution rate being equal to 73–80 μmol O_2_ (mg Chl h)^–1^ in both CC-503 and 137c cells. Such lag phase has been previously described for CO_2_-dependent O_2_ evolution in cyanobacteria [39] and, as proposed, could be due to the operation of the ETC participants located between PSII and PSI. After the addition of 0.5 mM FeCy to the algal cells, the curves of O_2_ evolution were similar to those observed in the absence of additions (Figure 3). Two-fold increases in FeCy concentration (up to 1.0 mM), since according to previous publications, 0.5 and 1.0 mM FeCy paired with DCBQ are often used in studies of *C. reinhardtii* cells [13,15,16], did not affect the slope of the observed O_2_ evolution curves. This clearly indicates that molecules of water-soluble FeCy were not able to penetrate through the plasma membrane into algal cells and did not affect the O_2_ evolution rate in living cells of *C. reinhardtii*.

In contrast, the addition of 0.1–0.2 mM DCBQ in the presence of 0.5 mM FeCy led to the immediate beginning of O_2_ evolution after actinic light was switched on in cells of both strains (Figure 3). This supports the possibility of DCBQ accepting electrons effectively from PSII in living algal cells, removing any possible limitations of electron transport caused by the ETC. Another point is the horizontal alignment of the dark-exposure part of the curve during the O_2_ evolution measurements, which had a slight decline in cells without additions and in the presence of FeCy. Both effects are consistent with those previously observed in cyanobacteria [39]. In addition, it was obvious that the cell wall had no influence on these effects.

### 3.3. Involvement of FeCy in the Action of DCBQ 

**Figure 4:**
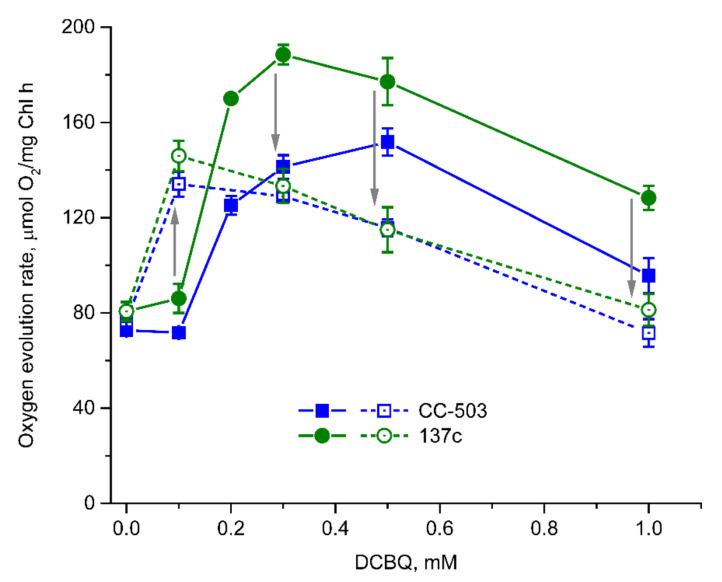
Dependence of the O_2_ evolution rate on DCBQ concentration in the absence of FeCy (solid lines) and after the addition of 0.5 mM FeCy to the same sample with repeated measurement of O_2_ evolution (dashed lines) in living cells of CC-503 (blue squares) and 137c (green circles) strains of *C. reinhardtii*. Arrows indicate the direction of changes that occurred after FeCy addition in repeated measurements of O_2_ evolution. SDs (*n* = 5) are shown as bars.

The use of DCBQ without the addition of FeCy in the case of cell suspensions of both strains led to significant changes in the dependence of the O_2_ evolution rate on DCBQ concentration (Figure 4) compared with that measured in the presence of FeCy (Figure 2A). This is in spite of the fact that FeCy is not able to enter cells, as shown above, and could perform its function only in extracellular space.

At 0.1 mM DCBQ, the O_2_ evolution rate was detected to be almost at the same level as that in the absence of additions (Figure 4), and this was significantly different from the observations in the presence of FeCy (increases of up to ~166% and ~179% in CC-503 and 137c cells, respectively (Figure 2A)). An increase in DCBQ concentration resulted in strong stimulation of O_2_ evolution in cells of both strains. Thus, the maximum values were achieved with higher concentrations of DCBQ than those in the presence of FeCy, and their absolute values were lower (Figure 4). In the case of CC-503 cells, the maximum O_2_ evolution rate was reached at 0.5 mM DCBQ, with an increase by ~108% compared with that in the absence of DCBQ (~35% lower than that obtained with FeCy) after the stepwise increases at 0.2 mM and 0.3 mM DCBQ. The maximum O_2_ evolution rate in the case of 137c cells was reached at 0.3 mM DCBQ, with an increase by ~132% (~26% lower than that obtained with FeCy). 

The further increase in DCBQ concentrations above that necessary for achieving the maximum values of O_2_ evolution resulted in its suppression in a similar manner as that observed in the presence of FeCy (Figure 4). At 1 mM DCBQ, the decreases in DCBQ-induced O_2_ evolution in CC-503 and 137c cells were ~71% and ~56%, respectively, compared with the DCBQ-induced maximum. Interestingly, the shapes of the obtained curves for cells of both strains were quite similar, with relatively wide peaks (Figure 4), resembling the curves observed for CC-503 cells in the presence of FeCy (Figure 2A).

To study the role of FeCy in the described effects directly, 0.5 mM FeCy was added to the samples that were already used for the measurements of O_2_ evolution in the presence of DCBQ alone, and the dependence on DCBQ concentration was re-recorded. Significant differences in the detected values of the O_2_ evolution rates were observed (Figure 4). Thus, at 0.1 mM DCBQ, the cells of both strains showed increases of ~86% and ~70% in O_2_ evolution in the case of CC-503 and 137c, respectively, compared with the values obtained during the first measurement (in the presence of DCBQ alone); these increases were ~74% and ~80% if compared with the values obtained at 0 mM DCBQ, with clear suppression of O_2_ evolution at higher DCBQ concentrations. At 1 mM DCBQ, the values of the O_2_ rate almost reached the values observed at 0 mM DCBQ. The shape of the curves was similar to that observed above under the simultaneous addition of DCBQ and FeCy to the cell suspensions (Figure 2A). The overall decrease in the values of the O_2_ evolution rates indicated by the second measurement can be explained by the partial photoinhibition of PSII in cells illuminated during the first measurement (in the presence of DCBQ alone) for a couple of minutes.

The obtained data clearly indicate that the presence of FeCy led to the induction of the inhibitory effect of DCBQ on the O_2_ evolution rate in living algal cells at 2–3 times lower concentrations than those used in the absence of FeCy, probably because DCBQ was maintained in oxidized form. This is in spite of the fact that molecules of DCBQ reduced by PSII should diffuse out of the cell to be oxidized by FeCy and then enter the cell again.

### 3.4. Light-Induced Reduction in DCBQ by Cells 

**Figure 5:**
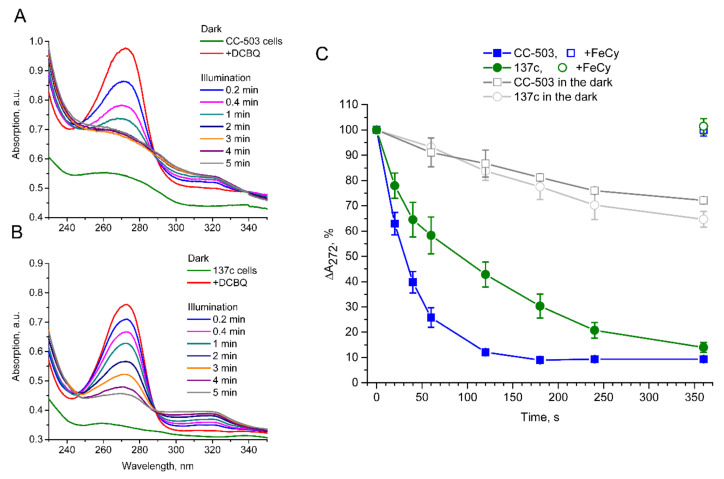
Light-induced reduction in DCBQ in the presence of living algal cells of *C. reinhardtii*. Panels (**A**,**B**), referring to the CC-503 and 137c, respectively, show the absorption changes in cell suspensions (green curve) under the addition of 30 µM DCBQ (red curve) and following illumination of the samples. The disappearance of the peak at 272–273 nm indicates DCBQ reduction over time. Panel (**C**) represents the absorption decrease at 272 nm under illumination in the case of CC-503 (blue-filled squares) and 137c (green-filled circles) cells over time. The unfilled blue square and green circle show the values after the addition of 0.2 mM FeCy. The gray unfilled squares and circles represent the CC-503 and 137c cells, respectively, after the addition of 30 µM DCBQ but without illumination. SDs (*n* = 3–4) are shown as bars.

To evaluate the influence of the presence of the cell wall on the diffusion efficiency of DCBQ, its reduction by PSII in CC-503 and 137c cells in the absence of FeCy was studied using the light-induced absorption changes related to the reduction in the oxidized form of DCBQ. As shown in Figure 5A,B, the addition of DCBQ led to the appearance of an intensive peak at 272–273 nm, which then diminished with the illumination of the samples, with the simultaneous appearance of relatively weak absorption in the 300–320 nm region, indicating the accumulation of DCBQ in reduced form. This is consistent with previously published results [35].

A significantly faster reduction in absorption at 272–273 nm under illumination in the case of CC-503 cells without a cell wall than in 137c cells with a well-formed cell wall was observed. The peak disappeared completely within 2 min in the CC-503 cell suspension (Figure 5A), while in the 137c cell suspension, this took much more time, and some absorption was observed even after 5 min of illumination (Figure 5B). The following calculations of the relative changes in absorption at 272 nm under illumination showed that 50% of the decrease was achieved in the presence of CC-503 cells during ~30 s of illumination, while it took ~90 s in the case of 137c cells (Figure 5C). Thus, the presence of the cell wall can lower the efficient DCBQ diffusion between extracellular and intracellular spaces by about three times.

At the same time, the presence of DCBQ in the cell suspension without the illumination of the samples resulted in a slight spontaneous decline of 7.3 ± 1.8% per min absorption at 272 nm (Figure 5C). In total, the decrease over 5 min was 28–35%. The results indicate the presence of a somehow light-independent weakly flowing reduction in DCBQ by *C. reinhardtii* cells, while this is accelerated greatly by photoinduced PSII action.

## 4. Discussion

The measurement of the maximum photoinduced O_2_ evolution by PSII in the presence of artificial acceptors is one of the commonly used methods for determining the photosynthetic apparatus state (or its change compared with a control, parent strain, etc.) in living cells of green algae because PSII is one of the most sensitive components of the thylakoid membrane. 

**Figure 6:**
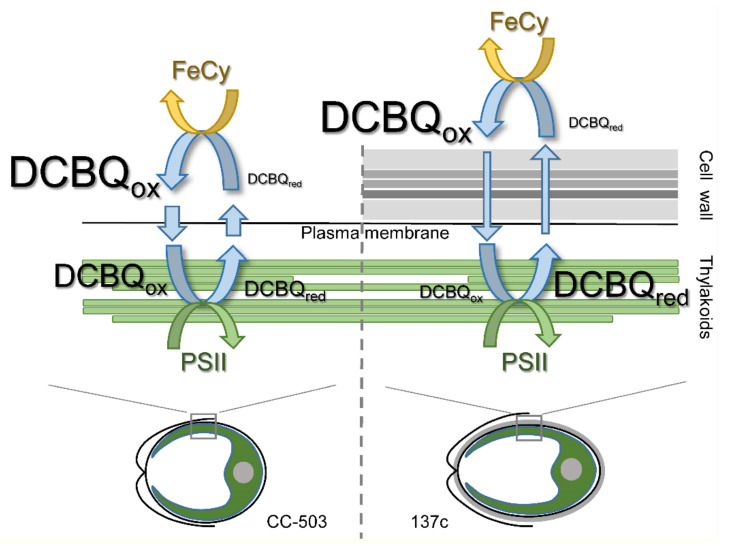
A scheme of the proposed cycle of DCBQ molecules diffusion in living cells of *C. reinhardtii* without (CC-503) and with (137c) a cell wall. Actions of PSII in the chloroplast and extracellular FeCy in the cycle are indicated by green and yellow arrows, respectively. The thickness of the straight blue arrows reflects the difference in diffusion efficiency of DCBQ molecules moving inside the cell and going back. The sizes of the “DCBQ” writing indicate possible differences between the contents of the oxidized (ox) and reduced (red) forms of DCBQ, respectively, inside and outside the algal cell.

The results obtained in the present study indicate that the presence of DCBQ paired with FeCy is indeed necessary to achieve the maximum O_2_ evolution rate by PSII in living *C. reinhardtii* cells. Thus, in the absence of FeCy, the maximum value of the O_2_ evolution rate in the presence of DCBQ was 17–22% lower (Figure 4) than the values detected under the addition of DCBQ together with FeCy (Figure 2A). The role of FeCy is thought to be to maintain DCBQ in the oxidized state (see, e.g., [2,8]), probably including the oxidation of molecules reduced by PSII. However, the comparison of the O_2_ evolution curves both in the absence of additions and in the presence of 0.5–1.0 mM FeCy (Figure 3) showed no differences, testifying that FeCy itself is not able to penetrate the plasma membrane of cells and thus can only act (i.e., oxidize DCBQ: DCBQ_red_→DCBQ_ox_) in the extracellular space. This is consistent with previously published data, which showed the inability of ascorbate located inside liposomes to reduce FeCy, while this reaction was significantly stimulated with the time constant (τ) of ~3.3 min by the addition of DCBQ, acting as a mediator [40]. Therefore, the molecules of DCBQ_ox_ that entered the algal cell and were reduced thereby PSII should diffuse out of the cell, where they can be oxidized by FeCy, and diffuse inside the cell again. Together, these processes form a cycle (Figure 6) in which the electroneutrality of reduced DCBQ molecules is maintained through protonation [41].

The effectiveness of such a cycle is strongly influenced by the presence of the cell wall since the photoinduced reduction in DCBQ_ox_ by PSII was ~3 times lower in the algal cells with a cell wall (Figure 5C), and the diffusion of DCBQ_ox_ from the extracellular space to PSII is obviously a main limiting factor. As a consequence, in cells with a cell wall, the balance between the oxidized and reduced (by PSII) forms of DCBQ should be shifted to the latter due to effective PSII operation (Figure 6), which, in turn, should lead to the decrease in the detected maximum values of the O_2_ evolution rate. However, as can be seen in Figure 2A, 137c cells with a cell wall showed higher values of maximum O_2_ evolution. Moreover, this was observed at 2–3 times lower concentrations of added DCBQ compared with CC-503 cells without a cell wall. A similar effect could also be seen even in the measurements conducted in the absence of FeCy (Figure 4). This discrepancy indicates a somehow inhibitory action of higher contents of the oxidized form of DCBQ on the O_2_-evolving activity of PSII.

The diffusion efficiency of DCBQ_ox_ in the case of cells without cell wall (CC-503) was higher (Figure 5) and could probably fully cover the rate of DCBQ reduction by PSII, which leads to establishing the DCBQ_ox_/DCBQ_red_ balance inside cells with equal or even higher content of DCBQ_ox_ (Figure 6). Based on the suggestion about the inhibitory action of DCBQ_ox_, this should result in the suppression of the O_2_ evolution activity of PSII to some extent. This can indeed be observed in the obtained experimental curves, especially in the absence of FeCy (Figure 4). At the same time, such inhibitory action of DCBQ_ox_ can weaken in the direction from the plasma membrane too deep into the cell due to the active continuous reduction in DCBQ_ox_ by PSII located above, decreasing the DCBQ_ox_ content for PSII located below. This can explain, for example, the wide peak of the maximum O_2_ evolution rate obtained for CC-503 cells (Figure 2A), which also appeared for 137c cells in the absence of FeCy (Figure 4) when the driving force of DCBQ oxidation was absent.

An increase in the concentration of added DCBQ should lead to a higher number of its molecules simultaneously diffusing through the plasma membrane of the cell. This is consistent, for example, with the previously obtained results of research on liposomes containing ascorbate, in which the τ of the FeCy reduction linearly decreased with the increase in DCBQ content [40]. The further increase in DCBQ concentration in the present study stimulated the inhibitory effect on the O_2_-evolving activity of PSII (Figure 2A and Figure 4). This clearly indicates the involvement of the oxidized form of DCBQ in this process because a much higher content of DCBQ_ox_ makes the contribution of PSII operation to the shift in the DCBQ_ox_/DCBQ_red_ balance insufficient. This is supported by data testifying that the reduction in the ability of DCBQ to be maintained in the oxidized form in the absence of FeCy in the mixture led to a significant weakening of the inhibitory effect of added DCBQ on the O_2_ evolution rate in living algal cells (Figure 2A and Figure 4). On the other hand, FeCy addition to the same samples induced strong suppression of O_2_ evolution by cells at DCBQ concentrations above 0.1 mM (Figure 4). FeCy addition at 0.1 mM DCBQ stimulated the O_2_ evolution rate in living algal cells, indicating the necessity of the presence of the DCBQ oxidation driving force at low DCBQ content for inducing maximum O_2_ evolution, as it was mentioned above. 

DCBQ is a quinone derivative with hydrophobic properties due to the presence of two Cl atoms [41], which allow it to have a high affinity for the Q_B_ site in PSII [6], where DCBQ can accept electrons directly from Q_A_. As it was previously calculated [6], the binding affinity of quinone derivatives increases with the extent of Cl substitution (by 25 times between monochloro-*p*-benzoquinone and tetrachloro-*p*-benzoquinone (chloranil)). In addition, the positions of Cl atoms in the molecule have a certain influence. Thus 2,6-DCBQ (DCBQ in the present study) has ~4 times higher affinity for the Q_B_ site than 2,5-DCBQ in the case of PSII preparations from cyanobacteria and ~6.7 times higher in the case of samples from higher plants [6,27].

In contrast to other artificial acceptors, the use of halogenated benzoquinones showed evidence of the presence of another (second) site of electrons accepting from PSII, which, as proposed, could be PQH_2_ molecules released from the Q_B_ site, for which 2,6- and 2,5-DCBQ have almost equal affinity [6,27]. As a consequence, the dependence of the O_2_ evolution rate on the concentration of different halogenated benzoquinones in PSII preparations from cyanobacteria and higher plants shows a non-straight line, with a significant decrease after a certain quinone concentration [6,27]. The same results were obtained in the present study in the suspensions of living *C. reinhardtii* cells with DCBQ as an acceptor (Figure 2 and Figure 4).

At the same time, the observed linear decrease in DCBQ_ox_ content (~7% per min) due to its reduction under dark incubation of the cell suspension (Figure 5C) allows us to propose the involvement of some minor enzyme of the thylakoid membrane in this process, for example, plastid terminal oxidase (PTOX). In vivo, PTOX reduces O_2_ to H_2_O due to PQH_2_ oxidation in higher plants and green algae [42,43], and it is obvious that DCBQ_ox_ reduction instead of O_2_ can be more favorable for the enzyme. This suggestion is supported by the observed horizontal alignment of the O_2_ evolution curve in the dark before the light-induced increase in the presence of DCBQ, which shows a slight decline in the absence of additions or in the presence of FeCy (Figure 3), probably due to the reduction in O_2_ by PTOX. The low content of PTOX (~1% from PSII components [42]) in the thylakoid membrane can explain the slow DCBQ reduction in the dark. 

According to the crystal structure of PSII, its acceptor side has a channel for the preplacement of the Q_B_ molecule, with an opening of ~10 per 20 Å on the membrane side. A cavity further provides a lipophilic environment favorable for the isoprenoid chain of Q_B_, as well as for rapid PQ/PQH_2_ exchange [44]. A recent study indicated that PQH_2_ is bound to the Q_B_ site ~50 times more weakly than PQ [45]. In addition, PQ^−^/PQH_2_ has a less positive value of the midpoint potential than PQ/PQ^−^ (~40 vs. ~90 mV, respectively). It is thought that these properties can be the driving force of PQH_2_ release into the PQ pool. A significant lowering of the potential was also found for halogenated benzoquinones between 1e^–^ and 2e^−^/2H^+^ reduction, respectively [46], which can cause changes in the degree of DCBQ binding and induces rapid DCBQ_red_-to-DCBQ_ox_ exchange in the Q_B_ site of PSII by analogy with the PQ/PQH_2_ exchange. However, unlike native Q_B_ (PQ B), which has a long isoprenoid chain (nine side units) capable of locking the channel for another Q_B_ molecule until release [44,47], the molecules of DCBQ are relatively small, which allows many of them in the oxidized form to enter to the cavity and tightly bind there at the same time. Based on the suggestions that DCBQ reduction should occur near Q_A_, i.e., deeper in the channel of the Q_B_ site, the release efficiency of a reduced molecule can be complicated by DCBQ_ox_ molecules bound tightly inside the cavity, and their number can be directly dependent on DCBQ_ox_ concentration near PSII. Thus, the increase in DCBQ_ox_ content near PSII suppresses the effectiveness of the DCBQ_red_¬-to-DCBQ_ox_ exchange, leading, in turn, to a decrease in the O_2_ evolution rate, which was clearly observed during experiments of the present study (Figure 2A and Figure 4).

The results obtained indicate the strong dependence of the maximum O_2_ evolution rate of PSII in living cells of *C. reinhardtii* on the DCBQ concentration and the presence of FeCy with significant suppression when using DCBQ concentrations higher than a certain threshold. It has to be exactly near 0.1 mM DCBQ in the case of algal cells with a cell wall and in the range of 0.2–0.4 mM DCBQ for cells without a cell wall. However, even in this case, the values obtained may be the result of a balance between the actual maximum O_2_ evolution rate and its partial inhibition by DCBQox, which should be taken into account by researchers in their studies.

## Data Availability

The datasets generated during and/or analyzed during the current study are available from the corresponding author upon reasonable request.

## References

[1] Müh F., Glöckner C., Hellmich J., Zouni A. (2012). Light-Induced Quinone Reduction in Photosystem II. Biochim. Biophys. Acta-Bioenerg..

[2] Virtanen O., Khorobrykh S., Tyystjärvi E. (2021). Acclimation of Chlamydomonas Reinhardtii to Extremely Strong Light. Photosynth. Res..

[3] Terentyev V.V., Shukshina A.K., Shitov A.V. (2019). Carbonic Anhydrase CAH3 Supports the Activity of Photosystem II under Increased PH. Biochim. Biophys. Acta-Bioenerg..

[4] Shevela D., Messinger J. (2012). Probing the Turnover Efficiency of Photosystem II Membrane Fragments with Different Electron Acceptors. Biochim. Biophys. Acta-Bioenerg..

[5] Suzuki T., Minagawa J., Tomo T., Sonoike K., Ohta H., Enami I. (2003). Binding and Functional Properties of the Extrinsic Proteins in Oxygen-Evolving Photosystem II Particle from a Green Alga, Chlamydomonas Reinhardtii Having His-Tagged CP47. Plant Cell Physiol..

[6] Satoh K., Oh-hashi M., Kashino Y., Koike H. (1995). Mechanism of Electron Flow through the QB Site in Photosystem II. 1. Kinetics of the Reduction of Electron Acceptors at the QB and Plastoquinone Sites in Photosystem II Particles from the Cyanobacterium Synechococcus Vulcanus. Plant Cell Physiol..

[7] Dudekula S., Fragata M. (2006). Investigation of the Electron Transfer Site of P-Benzoquinone in Isolated Photosystem II Particles and Thylakoid Membranes Using α- and β-Cyclodextrins. J. Photochem. Photobiol. B Biol..

[8] Yanykin D.V., Khorobrykh A.A., Khorobrykh S.A., Klimov V.V. (2010). Photoconsumption of Molecular Oxygen on Both Donor and Acceptor Sides of Photosystem II in Mn-Depleted Subchloroplast Membrane Fragments. Biochim. Biophys. Acta-Bioenerg..

[9] Biswas S., Eaton-Rye J.J. (2018). PsbY Is Required for Prevention of Photodamage to Photosystem II in a PsbM-Lacking Mutant of Synechocystis Sp. PCC 6803. Photosynthetica.

[10] Shitov A.V., Terentyev V.V., Zharmukhamedov S.K., Rodionova M.V., Karacan M., Karacan N., Klimov V.V., Allakhverdiev S.I. (2018). Is Carbonic Anhydrase Activity of Photosystem II Required for Its Maximum Electron Transport Rate?. Biochim. Biophys. Acta-Bioenerg..

[11] Rudenko N.N., Fedorchuk T.P., Terentyev V.V., Dymova O.V., Naydov I.A., Golovko T.K., Borisova-Mubarakshina M.M., Ivanov B.N. (2020). The Role of Carbonic Anhydrase α-CA4 in the Adaptive Reactions of Photosynthetic Apparatus: The Study with α-CA4 Knockout Plants. Protoplasma.

[12] Roach T., Sedoud A., Krieger-Liszkay A. (2013). Acetate in Mixotrophic Growth Medium Affects Photosystem II in Chlamydomonas Reinhardtii and Protects against Photoinhibition. Biochim. Biophys. Acta-Bioenerg..

[13] Hamilton M.L., Franco E., Deák Z., Schlodder E., Vass I., Nixon P.J. (2014). Investigating the Photoprotective Role of Cytochrome B-559 in Photosystem II in a Mutant with Altered Ligation of the Haem. Plant Cell Physiol..

[14] Nishimura T., Sato F., Ifuku K. (2017). In Vivo System for Analyzing the Function of the PsbP Protein Using Chlamydomonas Reinhardtii. Photosynth. Res..

[15] Virtanen O., Valev D., Kruse O., Wobbe L., Tyystjärvi E. (2019). Photoinhibition and Continuous Growth of the Wild-Type and a High-Light Tolerant Strain of Chlamydomonas Reinhardtii. Photosynthetica.

[16] Cecchin M., Jeong J., Son W., Kim M., Park S., Zuliani L., Cazzaniga S., Pompa A., Young Kang C., Bae S. (2021). LPA2 Protein Is Involved in Photosystem II Assembly in Chlamydomonas Reinhardtii. Plant J..

[17] Longatte G., Rappaport F., Wollman F.-A., Guille-Collignon M., Lemaître F. (2017). Electrochemical Harvesting of Photosynthetic Electrons from Unicellular Algae Population at the Preparative Scale by Using 2,6-Dichlorobenzoquinone. Electrochim. Acta.

[18] Sayegh A., Longatte G., Buriez O., Wollman F.-A., Guille-Collignon M., Labbé E., Delacotte J., Lemaître F. (2019). Diverting Photosynthetic Electrons from Suspensions of Chlamydomonas Reinhardtii Algae—New Insights Using an Electrochemical Well Device. Electrochim. Acta.

[19] Beauzamy L., Beauzamy L., Delacotte J., Bailleul B., Tanaka K., Nakanishi S., Wollman F.A., Lemaître F. (2020). Mediator-Microorganism Interaction in Microbial Solar Cell: A Fluo-Electrochemical Insight. Anal. Chem..

[20] Shlosberg Y., Schuster G., Adir N. (2022). Harnessing Photosynthesis to Produce Electricity Using Cyanobacteria, Green Algae, Seaweeds and Plants. Front. Plant Sci..

[21] Kirchhoff H. (2014). Diffusion of Molecules and Macromolecules in Thylakoid Membranes. Biochim. Biophys. Acta-Bioenerg..

[22] Terentyev V.V. (2021). Loss of Carbonic Anhydrase in the Thylakoid Lumen Causes Unusual Moderate-Light-Induced Rearrangement of the Chloroplast in Chlamydomonas Reinhardtii as a Way of Photosystem II Photoprotection. Plant Physiol. Biochem..

[23] Polukhina I., Fristedt R., Dinc E., Cardol P., Croce R. (2016). Carbon Supply and Photoacclimation Cross Talk in the Green Alga Chlamydomonas Reinhardtii. Plant Physiol..

[24] Jursinic P.A., Dennenberg R.J. (1988). Enhanced Oxygen Yields Caused by Double Turnovers of Photosystem II Induced by Dichlorobenzoquinone. Biochim. Biophys. Acta-Bioenerg..

[25] Halverson K.M., Barry B.A. (2003). Sucrose and Glycerol Effects on Photosystem II. Biophys. J..

[26] Iwai M., Katoh H., Katayama M., Ikeuchi M. (2004). PSII-Tc Protein Plays an Important Role in Dimerization of Photosystem II. Plant Cell Physiol..

[27] Kashino Y., Yamashita M., Okamoto Y., Koike H., Satoh K. (1996). Mechanisms of Electron Flow through the QB Site in Photosystem II. 3. Effects of the Presence of Membrane Structure on the Redox Reactions at the QB Site. Plant Cell Physiol..

[28] Goodenough U.W., Heuser J.E. (1985). The Chlamydomonas Cell Wall and Its Constituent Glycoproteins Analyzed by the Quick-Freeze, Deep-Etch Technique. J. Cell Biol..

[29] Baudelet P.-H., Ricochon G., Linder M., Muniglia L. (2017). A New Insight into Cell Walls of Chlorophyta. Algal Res..

[30] Shutova T., Kenneweg H., Buchta J., Nikitina J., Terentyev V., Chernyshov S., Andersson B., Allakhverdiev S.I., Klimov V.V., Dau H. (2008). The Photosystem II-Associated Cah3 in Chlamydomonas Enhances the O2 Evolution Rate by Proton Removal. EMBO J..

[31] Terentyev V.V., Shukshina A.K., Ashikhmin A.A., Tikhonov K.G., Shitov A.V. (2020). The Main Structural and Functional Characteristics of Photosystem-II-Enriched Membranes Isolated from Wild Type and Cia3 Mutant Chlamydomonas Reinhardtii. Life.

[32] Shukshina A.K., Terentyev V.V. (2021). Involvement of Carbonic Anhydrase CAH3 in the Structural and Functional Stabilization of the Water-Oxidizing Complex of Photosystem II from Chlamydomonas Reinhardtii. Biochemistry.

[33] Work V.H., Radakovits R., Jinkerson R.E., Meuser J.E., Elliott L.G., Vinyard D.J., Laurens L.M.L., Dismukes G.C., Posewitz M.C. (2010). Increased Lipid Accumulation in the Chlamydomonas Reinhardtii Sta7-10 Starchless Isoamylase Mutant and Increased Carbohydrate Synthesis in Complemented Strains. Eukaryot. Cell.

[34] Porra R.J., Thompson W.A., Kriedemann P.E. (1989). Determination of Accurate Extinction Coefficients and Simultaneous Equations for Assaying Chlorophylls a and b Extracted with Four Different Solvents: Verification of the Concentration of Chlorophyll Standards by Atomic Absorption Spectroscopy. Biochim. Biophys. Acta-Bioenerg..

[35] Semin B., Davletshina L.N., Rubin A.B. (2019). Effect of Sucrose-Bound Polynuclear Iron Oxyhydroxide Nanoparticles on the Efficiency of Electron Transport in the Photosystem II Membranes. Photosynth. Res..

[36] Cronmiller E., Toor D., Shao N.C., Kariyawasam T., Wang M.H., Lee J.-H. (2019). Cell Wall Integrity Signaling Regulates Cell Wall-Related Gene Expression in Chlamydomonas Reinhardtii. Sci. Rep..

[37] Shetty P., Gitau M.M., Maróti G. (2019). Salinity Stress Responses and Adaptation Mechanisms in Eukaryotic Green Microalgae. Cells.

[38] Iwai M., Roth M.S., Niyogi K.K. (2018). Subdiffraction-resolution Live-cell Imaging for Visualizing Thylakoid Membranes. Plant J..

[39] Samuilov V.D., Fedorenko T.A. (1999). Lag Phase of CO2-Dependent O2 Evolution by Illuminated Anabaena Variabilis Cells. Biochemistry.

[40] Ilani A., Krakover T. (1987). Diffusion- and Reaction Rate-Limited Redox Processes Mediated by Quinones through Bilayer Lipid Membranes. Biophys. J..

[41] Itoi H., Tazawa S., Hasegawa H., Tanabe Y., Iwata H., Ohzawa Y. (2019). Study of the Pore Structure and Size Effects on the Electrochemical Capacitor Behaviors of Porous Carbon/Quinone Derivative Hybrids. RSC Adv..

[42] Lennon A.M., Prommeenate P., Nixon P.J. (2003). Location, Expression and Orientation of the Putative Chlororespiratory Enzymes, Ndh and IMMUTANS, in Higher-Plant Plastids. Planta.

[43] Feilke K., Streb P., Cornic G., Perreau F., Kruk J., Krieger-Liszkay A. (2016). Effect of Chlamydomonas Plastid Terminal Oxidase 1 Expressed in Tobacco on Photosynthetic Electron Transfer. Plant J..

[44] Loll B., Kern J., Saenger W., Zouni A., Biesiadka J. (2005). Towards Complete Cofactor Arrangement in the 3.0 Å Resolution Structure of Photosystem II. Nature.

[45] De Causmaecker S., Douglass J.S., Fantuzzi A., Nitschke W., Rutherford A.W. (2019). Energetics of the Exchangeable Quinone, Q B, in Photosystem II. Proc. Natl. Acad. Sci. USA.

[46] Huynh M.T., Anson C.W., Cavell A.C., Stahl S.S., Hammes-Schiffer S. (2016). Quinone 1 e^−^ and 2 e^−^/2 H^+^ Reduction Potentials: Identification and Analysis of Deviations from Systematic Scaling Relationships. J. Am. Chem. Soc..

[47] Guskov A., Kern J., Gabdulkhakov A., Broser M., Zouni A., Saenger W. (2009). Cyanobacterial Photosystem II at 2.9-Å Resolution and the Role of Quinones, Lipids, Channels and Chloride. Nat. Struct. Mol. Biol..

